# Personality and Pain Outcomes in Rheumatic Disease: The Mediating Role of Psychological Flexibility

**DOI:** 10.3390/healthcare12111087

**Published:** 2024-05-25

**Authors:** Cristiana-Manuela Cojocaru, Cosmin Octavian Popa, Alina Schenk, Ștefan Marian, Horia Marchean, Bogdan Andrei Suciu, Simona Szasz, Horațiu Popoviciu, Simona Mureșan

**Affiliations:** 1 The Doctoral School, George Emil Palade University of Medicine, Pharmacy, Science and Technology, 540142 Targu-Mures, Romania; cristiana-manuela.cojocaru@umfst.ro (C.-M.C.); alina.muresan@umfst.ro (A.S.); horia.marchean@umfst.ro (H.M.); 2 Department of Ethics and Social Sciences, George Emil Palade University of Medicine, Pharmacy, Science and Technology, 540142 Targu-Mures, Romania; 3 Department of Psychology, West University of Timişoara, 300223 Timişoara, Romania; stefan.marian@e-uvt.ro; 4 Department of Anatomy and Morphological Science, George Emil Palade University of Medicine, Pharmacy, Science, and Technology, 540142 Targu-Mures, Romania; suciubogdanandrei@yahoo.com; 5 Department of Rheumatology, Physical and Rehabilitation Medicine, George Emil Palade University of Medicine, Pharmacy, Science and Technology, 540142 Targu-Mures, Romania; szasz_fc@yahoo.com (S.S.); horatiu.popoviciu@umfst.ro (H.P.); 6 Department of Internal Medicine IV, George Emil Palade University of Medicine, Pharmacy, Science and Technology, 540142 Targu-Mures, Romania; simona.muresan@umfst.ro

**Keywords:** chronic pain, personality, psychological flexibility, pain-related distress, psychological inflexibility

## Abstract

Background: Chronic pain is associated with increased disability and vulnerability to emotional disorders. Personality and psychological flexibility (PF) describe interindividual differences that shape the adjustment to chronic pain. Specifically, PF was found to be associated with pain, fatigue, anxiety, and depression intensity. Although previous studies established strong correlations between personality and pain outcomes, evidence on the nature of this relationship is scarce. Therefore, the objective of this study is to explore the mediating effect of PF on the relationship between personality and distress. Methods: This transversal study included 108 participants (age M = 56.7, SD = 11.3) diagnosed with musculoskeletal chronic pain. Self-reported measures were administered by the medical care team. Multiple mediation models were performed for estimating the indirect effects on each outcome variable. Results: After controlling for age and gender covariates, we found that PF completely mediated the relationship between personality traits and all pain outcomes and partially mediated the impact of extraversion on anxiety. In addition, emotional stability also had an indirect effect on anxiety through PF. Conclusions: Personality traits and PF are significant predictors of pain outcomes. PF represents a core process mediating the impact of personality traits on the perceived intensity of pain, fatigue, anxiety, and depression in patients with rheumatic disease. These results could facilitate the application of individualized psychological interventions in clinical contexts targeting the reduction of emotional avoidance and in chronic pain patients.

## 1. Introduction

Chronic pain is defined as pain that lasts for over three months in the absence of tissue damage, impacting both physical and emotional health [1]. The incidence of chronic pain over one year was estimated at 5.24% [2]. The prevalence rate has risen up to 30% in the general population lately, predominantly affecting women, who are more likely to report higher pain intensity and related stress levels [3]. Additionally, fatigue is present in over half of chronic pain patients, encompassing a subjective negative experience influenced by both physiological and psychological aspects [4]. Against this background, a significant number of patients with chronic pain develop emotional disorders, such as anxiety and depression, which co-occur in around 20–30% individuals [5]. Investigations of the frequent comorbidity between anxiety and depression indicate the existence of a network consisting of common symptoms, such as sad mood and concentration difficulties, emerging from underlying interacting elements that contribute to disorder onset. The transdiagnostic nature of these elements suggests the involvement of common risk factors explaining these interrelations [6,7]. Also, the overlap between neural activity patterns in chronic pain and emotional disorders shows several common particularities, like gray matter changes within the prefrontal cortex and insula, which indicates the presence of shared mechanisms that potentiate each other, paving the path toward chronicity across these diagnoses. Particularly, avoidance represents a proposed process thought to have a central function in this network, exacerbating dysfunctionality [8]. In turn, this might escalate into symptoms like anxiety, depression, sleeping problems, and fatigue, worsening the overall disturbance generated by chronic pain [6]. In this context, the transition from optimal functioning to psychopathology can be explained by the existence of two-sided processes that encompass both risk and protective factors [9,10].

Among the most prominent variables associated with the adjustment to chronic pain, several personality traits and psychological flexibility have been pointed out so far [11,12,13,14]. Personality traits are described as relatively stable patterns of behavior shaping individual differences [15]. The unique variabilities in their levels can be grouped into different profiles that may serve as predictors of multiple functional outcomes [16]. One of the most widely used approaches in the study of personality is the Five Factor Model (FFM), which includes five core traits, namely neuroticism (i.e., the opposite of emotional stability), extraversion, consciousness, openness, and agreeableness [17]. Neuroticism is regarded as a general risk factor for psychopathology, defined by the proneness to react more intensely to stress [18,19]. In relation to chronic pain, neuroticism is associated with distorted pain perception, fear of movement and disability [20]. Conversely, emotional stability refers to the ability to remain calm in demanding situations, such as living with illness, demonstrating increased capacity to adapt [21]. Extraversion (i.e., the preference for social interactions) correlates with positive emotions, approach motivation, as well as the use of healthy coping strategies in chronic pain [22]. In addition, consciousness (i.e., the tendency toward structure and discipline), openness to intellectual experiences (i.e., the capacity to associate various concepts, curiosity, and creativity), and agreeableness (i.e., interpersonal warmth, kindness, and cooperation) are thought to have rather indirect effects on health-related outcomes in chronic pain through different lifestyle choices and emotion management skills [22,23,24].

In parallel, psychological flexibility (PF) is described as the ability to adjust by acting in concordance with contextual features, comprising several important processes. Specifically, this involves an increased awareness of the present moment, along with a healthy way of dealing with sensations, emotions, and thoughts, including a realistic self-perception (i.e., the opposite of identifying oneself with the illness). Also, PF refers to the capacity to direct behaviors in the service of personal life values, despite daily hassles and difficult life circumstances. In contrast, psychological inflexibility (PI) means an inadequate way of relating to thoughts and feelings, along with engaging in dysfunctional behaviors that ultimately hinders one’s adjustment to unfavorable life events [25]. Experiential avoidance is a core process of PI, representing one’s attempt to prevent the experience of unpleasant emotions and/or physical sensations at the expense of general well-being [26]. Due to the centrality of experiential avoidance within this model and its influence on many functional variables, this process is now broadly recognized as a well-founded indicator of low PF, referring to a broad construct that has been proven to have close connections to other PI processes [27]. This dysfunctional process has been repeatedly proven to have strong correlations with various forms of psychopathology, including anxiety, depression, and psychotic disorders, constituting an independent construct focusing on avoidance as a transdiagnostic feature among these disorders [28,29,30,31]. In chronic pain, low PF was linked to higher pain impact and an increased risk of developing emotional disorders [32,33].

In the PF framework, avoidance behaviors like overuse of opioids or withdrawal from activities (e.g., relying on sick leave) are reinforced due to the short-term relief of pain and related unpleasant internal experiences [34]. Additionally, neuroticism is characterized by avoidance coping, which further accentuates stress reactivity [18]. In this way, PI can be considered as a transdiagnostic process linking dispositional features and pain outcomes through the unwillingness to experience noxious stimulation [35,36]. At the same time, high extraversion, consciousness, openness, and agreeableness are viewed as protective personality traits that promote better coping with pain-related difficulties [22,24]. Moreover, PF was found to mediate the relationship between personality and well-being in the general population. This emphasized its nature as a proximal and modifiable characteristic, in contrast to personality traits, which define more distal predictors of functioning [37]. Based on these results, we assume that the influence of personality traits on pain outcomes is carried over through PF, conceptualized as a learned life skill that may facilitate adjustment. As far as we know, this is the first study to investigate the role of PF/PI in the pathway from personality to pain outcomes. More exactly, the main objective of the present study is to test the mediating role of PF in the relationship between each personality trait (as conceptualized within the FFM) and common pain-related outcomes in chronic pain. The first hypothesis of the study is that low emotional stability, extraversion, conscientiousness, openness, agreeableness, and PF would correlate with high self-rated pain, fatigue, anxiety, and depression. The second hypothesis is that personality traits (i.e., emotional stability and neuroticism) and PF would predict pain, fatigue, anxiety, and depression. The third hypothesis is that PF would mediate the relationship between personality traits (i.e., emotional stability and extraversion) and pain outcomes.

## 2. Materials and Methods

### 2.1. Participants

**Figure 1:**
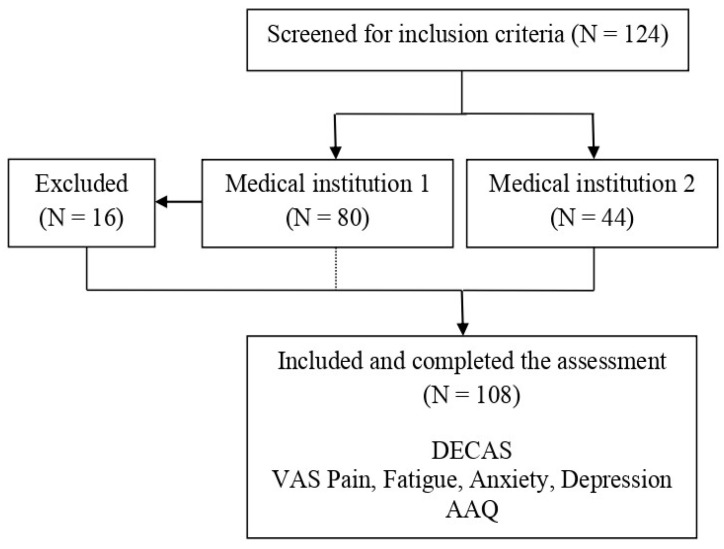
Flow chart of the study procedure.

The sample size was calculated using G-Power software, version 3.1, with the linear multiple regression statistical test, involving a fixed model, single regression coefficient, and a total number of six predictors. The input parameters were the following: medium effect size (f^2^ = 0.15), alpha error probability of 0.05, and power of 0.80. The convenience sampling method was applied, resulting in a total number of 108 participants with ages ranging from 26 to 80 (M = 56.7, SD = 11.3). Participants were recruited from two independent medical facilities that used a multimodal approach in the management of chronic pain, including medical treatment and physical and complementary therapies. More exactly, 64 questionnaires were collected from a rheumatology clinic located in Mures county, named the Apollo Wellness Club (medical institution 1), while 44 questionnaires were collected from the Rheumatology section of the Targu Mures County Emergency Clinical Hospital (medical institution 2). Among the total of 80 participants initially enrolled at the Apollo Wellness Club, 16 were excluded due to incomplete data and no contact possibility. Figure 1 depicts the flow chart of the study. The inclusion criteria were: (1) the presence of pain for at least 3 months within a formal diagnosis established by a rheumatologist; (2) minimum age of 18 years or over; and (3) comprehension of Romanian language. The exclusion criteria were: (1) acute pain episode; (2) diagnosis of severe psychiatric disorders established by a psychiatrist; and (3) serious cognitive impairment.

### 2.2. Ethical Considerations

This study was approved by the Ethics Committee for Scientific Research of the George Emil Palade University of Medicine, Pharmacy, Science and Technology, through an official decision issued on 22 July 2021, under number 1447. Participants received information regarding the objectives, methods, and procedures of the research and provided signed informed consent prior to enrollment.

### 2.3. Measures

The DECAS inventory [38] is a personality assessment tool validated on the Romanian population, relying on the FFM. The instrument comprises 95 items for measuring the dimensions of openness, extraversion, consciousness, agreeableness, and emotional stability (i.e., neuroticism if reversely scored), along with two additional control items. Respondents are asked to provide dichotomous answers by choosing if a specific statement is “true” or “false” for them, most of the time. Online software is used for interpreting individual answers, in the form of a cohesive profile, which is validated by the experimenter according to the normative sample based on gender and age groups. The instrument demonstrated acceptable reliability in the original study, with the internal consistency Cronbach’s alpha coefficients being 0.71 for openness and agreeableness, 0.75 for extraversion, 0.70 for consciousness, and 0.74 for emotional stability. Also, the DECAS tool demonstrated significant correlations with other instruments based on the FFM when comparing each subscale dimension [16].

A visual analog scale (VAS) was used for evaluating the intensity of distress variables. This measure consists of a unique item that comprises the main properties of the specific symptom of interest, requiring participants to answer by indicating the level of perceived discomfort on a scale from 0 to 10, with higher scores meaning increased severity. The utility of these brief subjective ratings is well documented in medical research, showing a reliable tool for assessing the intensity of pain and fatigue in patients with chronic conditions [39,40,41,42]. Although the VAS is not a common approach for investigating emotional distress, the use of this tool showed good convergent validity for measuring anxiety and depression through established correlations with other empirically validated instruments, with the obtained Pearson’s coefficients ranging between 0.60 and 0.74 [43,44]. In this research, participants were asked to rate the intensity of pain, fatigue, anxiety, and depression by referring to the previous two weeks. The scales used a horizontal format, including the following wording of anchors: 0 = “not at all”; 5 = “moderate”; and 10 = “severe”. Each pain outcome involved a corresponding item (e.g., “Over the last two weeks, how intense was the pain?”).

The Acceptance and Action Questionnaire (AAQ-II) [45] is a 7-item self-reported measure of PF. Higher scores represent lower levels of PF, equivalent to increased experiential avoidance, which is described as an unhealthy way of reacting to the subjective experience of uncomfortable emotions and sensations, typically resulting from the desire to control them. A 7-point Likert scale was used for rating the answers, starting with “never true” and ending with “always true”, with the total scores computed by summing up the individual items. The scale presented good psychometric properties on both clinical and healthy samples, as indicated by the Cronbach’s alpha internal consistency indicator of 0.84, as well as the predictive validity shown in the original validation study. The Romanian adaptation of the instrument is available, resembling the initial reliability level [46]. Also, in this study, the Cronbach’s alpha coefficient for AAQ was 0.83.

### 2.4. Design and Procedure

This study was conducted between 2021 and 2023 and employed an observational transverse design. The assessment protocol was composed of the DECAS personality inventory, the VAS for measuring subjective pain, fatigue, anxiety, and depression intensity, as well as the AAQ-II. The medical team formed by physicians, nurses, and a psychologist explained the instructions to each participant and administered the assessment protocol. Participants filled in the questionnaires during the waiting period before receiving medical treatment and/or a form of physical/complementary therapy. Afterward, the assessment protocols were collected by medical staff and delivered to other members of the research team, who oversaw the process of data collection and operation.

### 2.5. Statistical Analysis

**Figure 2:**
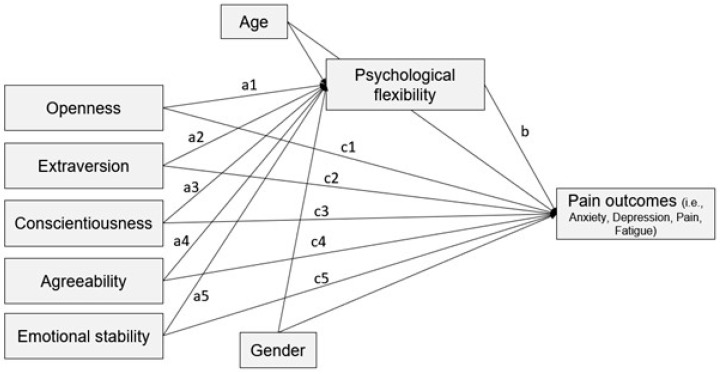
Hypothetical mediation model.

The first step of the statistical analysis consisted of exploring the relationships between all included variables, namely personality traits, PF, and pain outcomes. Every variable included in the analysis presented a normal distribution (−2 < skewness < 2 and −7 < kurtosis < 7); therefore, parametrical tests were selected. For this reason, Pearson’s two-tailed correlations were conducted. The second step involved testing the predictive role of personality traits and PF on each pain outcome by performing a multiple regression analysis. For verifying the normality and the constant variance of residuals, the function plot_model in the package sjPlot, version 2.8.15, was used. The histogram of residuals and the scatterplot of theoretical quartiles against standardized residuals were visually inspected, indicating data normality. For testing data homoscedasticity, a scatterplot of fitted values against residuals was visualized. As a third step, to test the mediation effect of PF on the relationship between personality traits and pain outcomes, we estimated four mediation models on manifest variables—one for each outcome variable (i.e., pain, fatigue, anxiety, and depression). Thus, each model included: (1) all five personality dimensions as predictors; (2) PF as mediator; and (3) one outcome variable at a time. The indirect effect for each separated personality trait, as well as the cumulative indirect effect from all personality traits at once, were investigated. For exploring the influence of potential confounding variables, age and gender were added as covariates in the mediation model. Figure 2 represents the hypothetical mediation model applied to each variable. The mediation analyses were performed using the package lavaan, version 0.6 [47], in R, version 4.3.2 [48].

## 3. Results

### 3.1. Demographic Characteristics

The demographic characteristics of the study sample are presented in Table 1. Most participants presented as the female gender, completed high school as their formal education, and were currently retired. The most frequent medical diagnosis was osteoarthritis, followed by cervical and/or lumbar spondylosis and rheumatoid arthritis.

### 3.2. Correlations

Descriptive statistics and the correlation matrix of all of the variables included in the mediation analyses can be found in Table 2. Significant positive correlations were found between personality traits, like openness and extraversion (r = 0.45), or agreeableness and emotional stability (r = 0.35). Additionally, as expected, low PF established negative correlations with personality traits, including openness (r = −0.32), extraversion (r = −0.38), and emotional stability (r = −0.46). Regarding the pain-related outcomes, negative correlations were found with personality traits, such as the association between reported pain severity and extraversion (r = −0.33) or between depression and emotional stability (r = −0.22). Conversely, positive correlations were shown with low PF for all outcome variables, namely pain (r = 0.39), fatigue (r = 0.35), anxiety (r = 0.55), and depression (r = 0.53).

### 3.3. Multiple Regression Models

The results of the multiple regression analyses are presented in Table 3. These results indicated direct relationships between predictors and outcome variables when not taking into account psychological flexibility. Specifically, psychological flexibility was significantly predicted by extraversion (t = −3.38, *p* = 0.02, 95% CI: −0.05, 0.00) and emotional stability (t = −3.27, *p* = 0.001, 95% CI: −0.08, −0.02). Pain was significantly predicted by extraversion (t = −3.04, *p* = 0.003, 95% CI: −0.14, −0.03) and agreeableness (t = −2.36, *p* = 0.02, 95% CI: −0.13, −0.01). Fatigue was significantly predicted by extraversion (t = −2.98, *p* = 0.003, 95% CI: −0.14, −0.03) and agreeableness (t = −2.36, *p* = 0.02, 95% CI: −0.12, −0.01). Additionally, extraversion significantly predicted anxiety (t = −3.00, *p* = 0.003, 95% CI: −0.15, −0.03) and depression (t = −3.43, *p* = 0.001, 95% CI: −0.15, −0.04). The inclusion of covariates into the regression analysis revealed a significant effect of age on pain (t = 2.03, *p* = 0.045, 95% CI: 0.00, 0.10) and fatigue (t = 2.12, *p* = 0.04, 95% CI: 0.00, 0.10). Also, gender was a significant predictor of pain (t = −2.61, *p* = 0.01, 95% CI: −3.15, −0.43), fatigue (t = −2.98, *p* = 0.003, 95% CI: −3.29, −0.66), and depression (t = −3.36, *p* = 0.001, 95% CI: −3.51, −0.90), but not anxiety.

### 3.4. Mediation Results

The results of the mediation analyses are presented in Table 4 and Table 5. Table 4 depicts the direct regression coefficients from the mediation models for direct paths (paths a, b, and c) and relationships between outcomes and covariates (age and gender). Table 5 presents the estimates for indirect effects and estimates for the total direct and indirect effects of the predictors and the mediator on each outcome variable.

In all tested models, we obtained a significant total indirect effect and an unsignificant direct effect indicating a full mediation of psychological flexibility on the relationship between personality traits and pain, fatigue, anxiety, and depression. However, only some individual mediation paths were significant. Specifically, PF significantly mediated the effect of extraversion (z = −0.01, *p* = 0.03, 95% CI: 0.01, −2.14) and emotional stability (z = −0.02, *p* = 0.005, 95% CI: 0.03, −2.83) on anxiety. In the case of the relationship between extraversion and anxiety, the direct path c remained significant, indicating a partial mediation, while the relationship between emotional stability and anxiety turned significant when including the mediator, even though direct paths a and b were unsignificant, indicating full mediation. Although only a few mediation paths were significant, including psychological flexibility as a mediator and a predictor for pain outcomes considerably improved the proportion of explained variance for all variables, ranging from 9% to 21%. 

## 4. Discussion

Relying on the previously proven impact of personality traits and PF on pain-related outcomes in medical conditions characterized by widespread musculoskeletal pain, the main purpose of the present study was to test the mediating effect of PF as a core modifiable process linked to adjustment in the path from personality traits to pain outcomes. Given the repeated associations found between emotional stability, extraversion, and pain-related outcomes, we expected that these personality traits would have an especially important role in predicting pain-related outcomes, in combination with PF. As stated in the first hypothesis, we found a pattern of negative correlations between personality traits, as well as between PF and pain outcomes. According to the second hypothesis, we found that personality traits and PF were significant predictors of pain outcomes. The third hypothesis was partially confirmed. After controlling for age and gender covariates, we obtained complete indirect effects of PF and personality traits for pain, fatigue, depression, and anxiety. In addition, PF was shown to mediate the impact of emotional stability and extraversion on anxiety, the latter path indicating a partial mediation. Overall, these findings matched with the psychological flexibility model, outlining the value of acceptance-based emotion regulation skills, instead of engaging in unproductive efforts to change emotional reactions and sensations for coping with challenging life situations such as facing chronic pain [34,49,50].

### 4.1. Correlations and Multiple Regression Results

The results of this study showed a strong association between personality traits, pain outcomes, and PF. First, we found positive correlations between openness, extraversion, and consciousness, as well as between emotional stability, extraversion, and agreeableness. This highlights the idea that personality constitutes an inclusive construct, composed of interrelated dimensions that potentiate each other. Particularly, extraversion, which is concerned with the overall activity level in relation to social interactions, stood out as an important personality feature that is directly proportional to the other areas of functioning in patients with chronic pain, resembling previous evidence found in the general population [51,52].

Second, openness and extraversion established a negative association with all pain outcomes. This is concordant with prior research documenting the co-occurrence between low extraversion, elevated pain intensity, and emotional interference [53,54]. These findings also emphasize the connection between personality and well-being. A potential mechanism explaining this relationship could be related to general behavioral attitudes, observed especially in social contexts [55]. In line with other results, extraversion and openness proved to have a negative correlation with pain and fatigue perception [56,57]. Nonetheless, emotional stability was specifically related to depression in our research. This is comparable to several analyses underlining that neuroticism as a tendency toward negative emotionality and intense stress reactivity is closely linked to the onset of emotional disorders [58,59]. In the same light, our results build on earlier conclusions regarding the connection between neuroticism and depression levels in chronic pain patients [60,61].

Third, PF was negatively correlated with openness, extraversion, agreeableness, and emotional stability. Likewise, past investigations showed that low PF is directly proportional with high neuroticism and psychopathological personality traits, indicating poor adjustment abilities [62]. Regarding the influence on pain perception, our results are comparable to other studies indicating that physical functioning is closely connected to PF, which proved to be a salient predictor of reported pain severity [63,64]. Also, we noticed a substantial association between PI and fatigue. Likewise, previous empirical evidence showed higher fatigue in patients was associated with a proneness to avoid unwanted internal experiences [65]. Also, in the same way as in our study, PI has been linked to anxiety and depressive symptoms, defining a dysfunctional way in which individuals react to emotions and physical sensations as part of the clinical picture of emotional disorders [66,67].

Fourth, the results of the multiple regression analyses pointed to the influence of age on pain and fatigue levels, as well gender on most pain outcomes, except anxiety. The impact of age on the physical interference resulting from chronic pain is understandable in the context of the aging process and gradually increasing vulnerability [68]. This outcome is concordant with past findings showing that women tend to present more negative consequences of pain than men, including physical and emotional symptoms [69,70]. Also, this could indicate that women are more prone to use emotional avoidance to cope with negative internal experiences, therefore demonstrating higher PI [71]. However, gender differences did not further impact the results of the mediation analyses in our study. This is also justifiable as PF could be conceptualized as a dynamic process encompassing a repertoire of emotion regulation skills that may promote an adequate response to various situational demands [72].

### 4.2. Results of the Mediation Analyses

In line with the psychological flexibility model, the mediation analyses demonstrated a substantial effect of PF on the relationship between personality traits and pain outcomes, regardless of age and gender covariates. Overall, the results of this research are well founded in the literature asserting that low PF might be a central process linking pain to clinical emotional symptoms and problematic functioning [73,74]. Regarding the impact of personality on pain perception, we found that the path from personality traits to pain intensity was mediated by PF. This is concordant with other investigations underlining the role of PF in shaping pain perception, especially in the context of preexisting vulnerability factors [73,75]. Moreover, the indirect effect of PF was preserved for the effects of personality traits on fatigue, suggesting that their combined impact on the physical components of well-being was stronger than when factors were treated individually [74]. Ultimately, our findings highlight that personal tendencies have an impact on fatigue levels through PF, which is similar to other conclusions pointing to the influence of these processes on subjective energy levels, above and beyond physical constraints [76,77].

Moreover, personality traits demonstrated an indirect effect on depression through PF. This indicates that the combination of personal tendencies linked to an increased frequency of negative effects and increased rigidity related to an inadequate way of managing these emotions represents a precursor for depressive symptoms in chronic pain [78,79]. In line with the psychological flexibility model, this accentuates the role of avoidance in the maintenance of depression in comorbidity with chronic pain by preventing the pursuit of meaningful life goals [80,81].

Interestingly, extraversion and emotional stability demonstrated an indirect effect mediated through PF only on anxiety in the present study. We believe this result is tenable given that the use of avoidance as a main coping mechanism for reducing somatic and/or emotional discomfort is a main feature of anxiety, extending beyond illness-related physical constraints [82,83]. In conjunction with temperamental tendencies defined by inhibition, which is a hallmark of neuroticism, as well as the tendency to withdraw from social exposure defining introversion, PI could intensify these stable individual patterns, thus contributing to anxiety [84]. For example, a person with a rheumatic diagnosis might withdraw from social interactions and other valued activities and engage in unhealthy ways of dealing with pain, which would further exacerbate association apprehension. In this way, failing to accept negative sensations and feelings could predispose the individual to develop anxiety disorders [85]. On the other hand, in the face of demanding and stressful life events, PF might act as a buffer by increasing one’s openness and willingness to embrace their aspirations despite unpleasant internal experiences [86].

Nonetheless, there are several constraints that may limit the interpretability of our results. First, the sample characteristics included wide variability in terms of age groups as well as a higher representation of the female gender based on the prevalence of chronic pain diagnoses in women. Second, we did not include a control group for comparing whether the same pattern of findings would be preserved for participants with different medical conditions, psychopathologies, or healthy individuals. Third, the cross-sectional nature of this research did not allow for assessing the potential modifications of PF and pain outcomes at different time points, along with the dynamics of their relationship. The inclusion of multiple measures at various intervals could inform whether personality traits and PF prove to be consistent predictors of pain, fatigue, anxiety, and depression. Also, replication of the mediation analysis would permit the formulation of firm conclusions regarding the role of avoidance and related dysfunctional processes in the relationship between personality traits and clinical symptoms. Fourth, all evaluations conducted in the present study relied on self-reported measures. For this reason, our findings could involve a significant amount of subjectivity and the provision of socially desirable answers. The use of multiple assessment tools, including physiological measures and clinician-rated scales, would increase data objectivity and accuracy. In this way, future studies could investigate the relationship between personality, PF, and physical or emotional interference in chronic pain using larger sample sizes and including participants belonging to multiple groups, as well as longitudinal assessments of state variables, especially pain, fatigue, anxiety, and depression.

### 4.3. Clinical Implications

Along with the investigation of biomedical characteristics, the identification of psychological particularities, including beliefs, expectations, and behavioral patterns, constitutes an integral part of a comprehensive clinical assessment in chronic pain [87]. By emphasizing the influence of personality traits and PF on pain outcomes, the present study could serve as a framework for including the evaluation of dispositional characteristics and psychological processes within a thorough intake assessment in chronic pain. This could also be a basis for designing more tailored instruments to address the diverse patient profiles in chronic pain, depending on the level of each personality trait and PF. Nevertheless, stereotyping and assigning patients to rigid delineated categories could be prevented by using a dimensional approach throughout the evaluation. In other words, acknowledging that personality traits and PF exist on spectrum, from low to high scores, could provide an exhaustive clinical presentation, drawing a nuanced and gradual line between accentuated dysfunctional features and psychopathology [84,88]. Also, the combined effect of personality traits and PF on pain outcomes highlights the need to focus more on the global presentation than on specific scores within clinical practice. For instance, a patient with chronic pain might present low emotional stability but high extraversion and PF, which could function as protective factors that facilitate psychological adjustment. Additionally, the assessment of personality features along with dysfunctional processes involved in psychopathology could inform the development and implementation of targeted interventions within an idiographic approach to pain management that considers interindividual heterogeneity when building the treatment plan [89]. This idea is founded in the process-based approach for psychological treatments aiming to improve PF [90]. In contrast to psychological interventions focused on symptom reduction, process-based psychotherapy works on replacing dysfunctional psychological processes with their functional counterparts, enabling emotion regulation and facilitating adjustment in the long run [91]. Furthermore, the role of PI in the relationship between personality traits and pain outcomes accentuates the need to integrate psychological approaches that specifically focus on improving PF as a necessary component of a multimodal rehabilitation plan for chronic pain, besides medical and physical therapies [92]. Hence, based on the individual profile, complementary non-pharmacological interventions could be flexibly adapted for maximizing benefits, such as providing treatments in a group format [93]. Ultimately, addressing the specific needs of different patient categories could enhance the healthcare quality of patients with rheumatic conditions involving chronic pain and emotional comorbidities, thereby facilitating long-term increases in their quality of life.

## 5. Conclusions

This study highlighted the mediating role of PF in the relationship between personality traits and pain-related outcomes in rheumatic disease. Anxiety was the only outcome for which the indirect effects of extraversion and emotional stability as individual paths were carried over through PF. The identification of these effects might facilitate the development of personalized care paradigms involving interdisciplinary treatment components in chronic pain aiming to increase PF for promoting better adjustment. In this context, the approaches used for psychological assessment and intervention in chronic pain could embrace a dimensional, process-based framework to enable long-term benefits.

## Figures and Tables

**Table 1:** Demographic characteristics of the study sample.

	Overall (N = 108)
Age	
Mean (SD)	56.7 (11.3)
Gender (N, %)	
Female	83 (76.9%)
Male	25 (23.1%)
Marital status	
Divorced	7 (6.5%)
Married	89 (82.4%)
Single	4 (3.7%)
Widowed	8 (7.4%)
Education	
High school	40 (37.0%)
Higher education	32 (29.6%)
Middle school	36 (33.3%)
Occupational status	
Employed	47 (43.5%)
Retired	50 (46.3%)
Unemployed	11 (10.2%)
Diagnosis	
Chronic post-surgical pain	4 (3.7%)
Coxarthrosis	3 (2.8%)
Gonarthrosis	4 (3.7%)
Osteoarthritis	30 (27.8%)
Other arthritis types	4 (3.7%)
Rheumatoid arthritis	24 (22.2%)
Rheumatoid polyarthritis	10 (9.3%)
Spondylosis	29 (26.9%)

**Table 2:** Pearson’s two-tailed correlations between personality, psychological flexibility, and pain outcomes.

Variable	M	SD	Sk	Ku	1	2	3	4	5	6	7	8	9
1. Openness	47.55	11.11	−0.33	0.08									
2. Extraversion	51.66	11.35	0.22	−0.42	0.45 **								
3. Conscientiousness	47.34	9.36	−0.36	0.44	0.23 *	0.13							
4. Agreeableness	49.36	10.23	0.23	0.50	−0.02	−0.16	−0.18						
5. Emotional Stability	47.22	8.45	0.20	−0.43	0.09	0.23 *	−0.14	0.35 **					
6. AAQ	2.91	1.31	0.72	0.18	−0.32 **	−0.38 **	−0.03	−0.21 *	−0.46 **				
7. Pain	5.70	3.05	−0.23	−0.98	−0.20 *	−0.33 **	−0.15	−0.12	−0.15	0.39 **			
8. Fatigue	5.93	2.94	−0.23	−1.05	−0.22 *	−0.32 **	−0.12	−0.09	−0.05	0.35 **	0.79 **		
9. Anxiety	4.12	3.15	0.54	−0.98	−0.30 **	−0.39 **	−0.10	−0.03	−0.17	0.55 **	0.59 **	0.60 **	
10. Depression	3.81	3.11	0.69	−0.75	−0.32 **	−0.42 **	−0.11	−0.07	−0.22 *	0.53 **	0.67 **	0.68 **	0.66 **

Note. ** *p* < 0.01, * *p* < 0.05, M = mean, SD = standard deviation, Sk = skewness, Ku = kurtosis. Abbreviations: AAQ—Acceptance and Action Questionnaire.

**Table 3:** Multiple regressions.

Criteria	Predictor	B	SE	t	*p*	[95% CI]		F(df)	r_adj_^2^
						LL	UL		
Psychological Flexibility	Intercept	7.93	1.14	6.96	**<0.001**	5.67	10.19	8.36 (7, 100) *p* < 0.001	0.32
	O	−0.02	0.01	−1.70	0.09	−0.04	0.00		
	E	−0.03	0.01	−2.38	**0.02**	−0.05	0.00		
	C	0.00	0.01	−0.24	0.81	−0.03	0.02		
	A	−0.02	0.01	−1.80	0.07	−0.04	0.00		
	S	−0.05	0.01	−3.27	**0.001**	−0.08	−0.02		
	Age	−0.52	0.27	−1.96	0.053	−1.05	0.01		
	Gender	0.01	0.01	1.38	0.17	−0.01	0.03		
Pain	Intercept	12.30	2.93	4.21	**<0.001**	6.50	18.11	4.47 (7, 100) *p* < 0.001	0.19
	O	0.00	0.03	0.02	0.98	−0.06	0.06		
	E	−0.09	0.03	−3.04	0.003	−0.14	−0.03		
	C	−0.04	0.03	−1.29	0.20	−0.10	0.02		
	A	−0.07	0.03	−2.36	**0.02**	−0.13	−0.01		
	S	0.02	0.04	0.44	0.66	−0.06	0.09		
	Age	0.05	0.02	2.03	**0.045**	0.00	0.10		
	Sex	−1.79	0.68	−2.61	**0.01**	−3.15	−0.43		
Fatigue	Intercept	9.87	2.83	3.49	<0.001	4.26	15.48	4.39 (7, 100) *p* < 0.001	0.18
	O	−0.01	0.03	−0.42	0.68	−0.07	0.04		
	E	−0.08	0.03	−2.98	0.003	−0.14	−0.03		
	C	−0.02	0.03	−0.65	0.52	−0.08	0.04		
	A	−0.07	0.03	−2.36	0.02	−0.12	−0.01		
	S	0.06	0.04	1.60	0.11	−0.01	0.13		
	Age	0.05	0.02	2.12	0.04	0.00	0.10		
	Gender	−1.97	0.66	−2.98	0.003	−3.29	−0.66		
Anxiety	Intercept	11.41	3.06	3.73	**<0.001**	5.34	17.47	3.99 (7, 100) *p* < 0.001	0.16
	O	−0.03	0.03	−1.18	0.24	−0.09	0.02		
	E	−0.09	0.03	−3.00	**0.003**	−0.15	−0.03		
	C	−0.01	0.03	−0.28	0.78	−0.07	0.05		
	A	−0.03	0.03	−1.00	0.32	−0.09	0.03		
	S	0.00	0.04	−0.11	0.92	−0.08	0.07		
	Age	0.03	0.03	1.00	0.32	−0.03	0.08		
	Gender	−1.32	0.72	−1.85	0.07	−2.74	0.10		
Depression	Intercept	11.23	2.81	3.99	**<0.001**	5.65	16.80	6.87 (7, 100) *p* < 0.001	0.28
	O	−0.03	0.03	−1.17	0.25	−0.09	0.02		
	E	−0.09	0.03	−3.43	**<0.001**	−0.15	−0.04		
	C	−0.01	0.03	−0.38	0.70	−0.07	0.05		
	A	−0.05	0.03	−1.61	0.11	−0.10	0.01		
	S	−0.01	0.04	−0.22	0.83	−0.08	0.06		
	Age	0.05	0.02	1.97	0.051	0.00	0.09		
	Gender	−2.21	0.66	−3.36	**0.001**	−3.51	−0.90		

Note. N = 108; B—unstandardized regression coefficient, SE—standard error, t—t-value, *p*—*p*-value (significant results are bolded), r_adj_^2^—adjusted r squared. Abbreviations: O—openness, E—extraversion, C—conscientiousness, A—agreeableness, S—emotional stability.

**Table 4:** Regression coefficients depicting direct relationships from mediation models.

Criteria	Predictor	B	SE	Z	*p*	[95% CI]		F(df)	r_adj_^2^
						LL	UL		
AAQ	O	−0.02	0.01	−1.77	0.08	−0.04	0.002	8.36 (7, 100) *p* < 0.001	0.33
	E	−0.03	0.01	−2.47	**0.01**	−0.05	−0.01		
	C	0.00	0.01	−0.25	0.81	−0.03	0.02		
	A	−0.02	0.01	−1.87	0.06	−0.04	0.001		
	S	−0.05	0.01	−3.40	**<0.001**	−0.07	−0.02		
	Age	0.01	0.01	1.43	0.15	−0.01	0.03		
	Sex	−0.52	0.26	−2.03	**0.04**	−1.02	−0.02		
Pain	AAQ	0.57	0.24	2.36	**0.02**	0.10	1.04	4.72 (8, 99) *p* < 0.001	0.22
	O	0.01	0.03	0.42	0.68	−0.04	0.06		
	E	−0.07	0.03	−2.61	**0.01**	−0.13	−0.02		
	C	−0.04	0.03	−1.32	0.19	−0.09	0.02		
	A	−0.06	0.03	−2.05	**0.04**	−0.11	−0.003		
	S	0.04	0.04	1.18	0.24	−0.03	0.12		
	Age	0.04	0.02	1.82	0.07	−0.003	0.09		
	Sex	−1.49	0.66	−2.28	**0.02**	−2.77	−0.21		
Fatigue	AAQ	0.55	0.23	2.35	**0.02**	0.09	1.00	4.63 (8, 99) *p* < 0.001	0.21
	O	0.00	0.03	−0.05	0.96	−0.05	0.05		
	E	−0.07	0.03	−2.54	**0.01**	−0.12	−0.02		
	C	−0.02	0.03	−0.63	0.53	−0.07	0.04		
	A	−0.06	0.03	−2.06	**0.04**	−0.11	0.00		
	S	0.08	0.04	2.35	**0.02**	0.01	0.15		
	Age	0.04	0.02	1.92	0.06	0.00	0.09		
	Sex	−1.69	0.63	−2.67	**0.01**	−2.93	−0.45		
Anxiety	AAQ	1.17	0.23	5.05	**<0.001**	0.72	1.63	7.20 (8, 99) *p* < 0.001	0.32
	O	−0.01	0.03	−0.50	0.62	−0.06	0.04		
	E	−0.06	0.03	−2.21	**0.03**	−0.11	−0.01		
	C	−0.01	0.03	−0.21	0.84	−0.06	0.05		
	A	−0.01	0.03	−0.24	0.81	−0.06	0.05		
	S	0.05	0.04	1.45	0.15	−0.02	0.12		
	Age	0.01	0.02	0.45	0.65	−0.03	0.05		
	Sex	−0.71	0.63	−1.12	0.26	−1.95	0.53		
Depression	AAQ	0.87	0.22	3.93	**<0.001**	0.44	1.31	8.56 (8, 99) *p* < 0.001	0.36
	O	−0.02	0.03	−0.62	0.54	−0.06	0.03		
	E	−0.07	0.03	−2.80	**0.01**	−0.12	−0.02		
	C	−0.01	0.03	−0.33	0.74	−0.06	0.04		
	A	−0.03	0.03	−1.06	0.29	−0.08	0.02		
	S	0.03	0.03	0.99	0.32	−0.03	0.10		
	Age	0.04	0.02	1.63	0.10	−0.01	0.08		
	Sex	−1.75	0.60	−2.91	**<0.001**	−2.94	−0.57		

Note. N = 108; B—unstandardized regression coefficient, SE—standard error, z—z-value, *p*—*p*-value (significant results are bolded), r_adj_^2^—adjusted r squared. Abbreviations: O—openness, E—extraversion, C—conscientiousness, A—agreeableness, S—emotional stability, AAQ—Acceptance and Action Questionnaire.

**Table 5:** Mediation results.

	Path	B	SE	z	*p*	95% CI	
						LL	UL
Pain r^2^ = 0.28 Δr^2^ = 0.06	O → AAQ → Pain	−0.01	−0.01	0.01	0.49	0.01	−0.70
	E → AAQ → Pain	−0.01	−0.02	0.01	0.48	0.01	−0.72
	C → AAQ → Pain	−0.01	−0.01	0.01	0.73	0.01	−0.35
	A → AAQ → Pain	−0.01	−0.01	0.01	0.50	0.01	−0.67
	S → AAQ → Pain	−0.01	−0.04	0.02	0.46	0.01	- 0.73
	Total indirect	−0.13	−0.22	−0.04	**0.01**	0.05	−2.70
	Total direct	−0.08	−0.21	0.06	0.26	0.07	−1.13
	Total effect	−0.20	−0.31	−0.10	**<0.001**	0.05	−3.83
Fatigue r^2^ = 0.27 Δr^2^ = 0.06	O → AAQ → Fatigue	−0.01	−0.02	0.01	0.20	0.01	−1.30
	E → AAQ → Fatigue	−0.01	−0.03	0.01	0.15	0.01	−1.43
	C → AAQ → Fatigue	−0.01	−0.01	0.01	0.70	0.01	−0.38
	A → AAQ → Fatigue	−0.01	−0.02	0.01	0.25	0.01	−1.16
	S → AAQ → Fatigue	−0.02	−0.05	0.01	0.11	0.01	−1.59
	Total indirect	−0.11	−0.20	−0.02	**0.02**	0.05	−2.39
	Total direct	−0.04	−0.17	0.01	0.53	0.07	−0.63
	Total effect	−0.15	−0.25	−0.05	**0.003**	0.05	−2.93
Anxiety r^2^ = 0.37 Δr^2^ = 0.05	O → AAQ → Anxiety	−0.02	−0.04	0.01	0.08	0.01	−1.76
	E → AAQ → Anxiety	−0.03	−0.05	−0.01	**0.03**	0.01	−2.14
	C → AAQ → Anxiety	−0.01	−0.03	0.02	0.70	0.01	−0.39
	A → AAQ → Anxiety	−0.02	−0.04	0.01	0.15	0.01	−1.45
	S → AAQ → Anxiety	−0.05	−0.08	−0.02	**0.005**	0.03	−2.83
	Total indirect	−0.18	−0.27	−0.08	**<0.001**	0.05	−3.70
	Total direct	−0.01	−0.14	0.11	0.83	0.06	−0.24
	Total effect	−0.19	−0.29	−0.09	**<0.001**	0.05	−3.58
Depression r^2^ = 0.41 Δr^2^ = 0.13	O → AAQ → Depression	−0.01	−0.02	0.01	0.16	0.01	−1.41
	E → AAQ → Depression	−0.01	−0.03	0.01	0.11	0.01	−1.60
	C → AAQ → Depression	−0.01	−0.01	0.01	0.70	0.01	−0.38
	A → AAQ → Depression	−0.01	−0.02	0.01	0.22	0.01	−1.24
	S → AAQ → Depression	−0.03	−0.05	0.01	0.07	0.01	−1.82
	Total indirect	−0.17	−0.26	−0.08	**<0.001**	0.05	−3.72
	Total direct	−0.06	−0.18	0.07	0.38	0.06	−0.88
	Total effect	−0.22	−0.32	−0.13	**<0.001**	0.05	−4.42

Note. r squared for the total effect; B—unstandardized regression coefficient, SE—standard error, z—z-value, *p*—*p*-value (significant results are bolded). Abbreviations: O—openness, E—extraversion, C—conscientiousness, A—agreeableness, S—emotional stability, AAQ—Acceptance and Action Questionnaire.

## Data Availability

The data presented in this study are available upon request from the corresponding author (cosmin.popa@umfst.ro).
